# Ferrimagnetic Tb/Co multilayers patterned by ion bombardment as substrates for magnetophoresis

**DOI:** 10.1038/s41598-024-73203-3

**Published:** 2024-10-10

**Authors:** Maciej Urbaniak, Daniel Kiphart, Michał Matczak, Feliks Stobiecki, Gabriel David Chaves-O’Flynn, Piotr Kuświk

**Affiliations:** Institute of Molecular Physics, Polish Academy of Sciences, Mariana Smoluchowskiego, 17 60-179 Poznań, Poland

**Keywords:** Fluidics, Surfaces, interfaces and thin films, Magnetic properties and materials

## Abstract

Ion bombardment with 30 keV Ga^+^ ions can locally change the magnetic properties of perpendicular magnetic anisotropy ferrimagnetic Tb/Co based multilayers. The induced changes in the effective magnetization create high gradients of magnetic fields in the proximity of the perimeters of the bombarded areas. Superparamagnetic, micrometer-sized beads floating in an aqueous suspension over such a patterned structure respond to the ensuing magnetostatic energy landscape. This landscape, and its associated forces on the beads, can be controlled with a time varying, external, homogeneous magnetic field. It is shown that with a 3.37 kA/m (approx. 4.2 mT) field and switching the direction at 10 Hz frequencies the beads can be driven with average forward velocities reaching 40 $\mu$m/s. This has the potential for use in lab-on-a-chip type assays.

**Supplementary Information:**

The online version contains supplementary material available at 10.1038/s41598-024-73203-3.

## Introduction

Although not widely accepted at the time, the first realization that certain minuscule creatures invisible to the naked eye can cause serious disease is at least 2050 years old^1^. Still, even now, societies worldwide constantly face new challenges related to diverse pathogens in spite of the astounding development of medical sciences in the past several hundred years. Highly increased mobility of people and international trade facilitate the spread of diseases in general^2^ and human impact on climate may result in a higher prevalence of fungal infections^3^, to name just two examples which adumbrate the next pandemic challenge. In any case, the control of diseases requires means of detection of pathogens, or even better, molecular typing techniques^4^ which can thoroughly characterize them. Most advanced methods require laboratory settings, but in recent years, the common experience of billions of people around the world has shown that relatively simple, rapid tests^5^ involving samples of nasal secretions, saliva, blood, etc., can make a huge difference in managing the epidemic response of government agencies. Moreover, these tests can often be self-administered by any individual (whether symptomatic or not) without the need to engage healthcare workers. The detection method usually relies on selective binding of reagents with the biomarker of a pathogen^6^ and such rapid tests can provide a result within 15–30 min ^7^, allowing for early self-isolation at home^5^.

Currently all established pathogen detection schemes, disregarding microscopy based methods, rely on the use of certain chemicals to induce a detectable signal (optical, electrical, etc.) in a sensor area/volume. Emerging remote techniques, such as Raman spectroscopy based detection^8^, while very promising, are not apposite to the present paper. In virtually all lab-on-a-chip type tests, i.e. those that can be performed outside of a laboratory setting, at least some of the reactants involved in the detection are transported to the reaction/detection site. Out of numerous detection strategies available, at least some involve the measurement of a magnetic field produced within the volume of the sensor by magnetic particles that connect to it by bridges containing sought-after antibodies^6^. The reactants can be mixed manually but the transport often involves capillary flow in an affordable, patterned cellulosic paper ^9^ or, in more elaborate schemes, can be performed in microfluidic devices^10–12^. In many of these devices^13^ a magnetic field is used to guide superparamagnetic beads^14^ (SPBs) which are surface functionalized to bind to the biomarkers of choice^15^. Traditionally, and in existing commercial products, the field is usually produced by macroscopic permanent magnets and/or electromagnets ^16,17^. In the last twenty years however, there is an increasing interest in utilizing the magnetostatic fields of patterned thin film structures, $H_{\textrm{str}}$, usually having an in-plane magnetic anisotropy, to locally produce high field gradients which are usable in microfluidic devices^18–21^. The gradients are high enough^22^ to attain average magnetophoretic velocities of SPBs reaching 0.3 mm/s^19,23^. We have shown in our previous papers that films with perpendicular magnetic anisotropy (PMA), such as sputtered Co/Au^24^ and Ir/Co^25^ systems can also be used as magnetophoretic substrates. Meanwhile, in a series of papers (see Ref.^26^ and references therein), Co/Au multilayers (MLs) patterned with energetic He$^+$ ions have been proven suitable for topologically protected, simultaneous, and independent transport of SPBs over various inhomogeneous energy landscapes. Although the established microfluidic systems can transport particles at velocities close to 10 mm/s^27^, i.e. 2 to 3 orders of magnitude higher than in our system, the use of the local magnetic energy landscape, like in our experiments, allows for a selective and simultaneous synthesis of colloidal bipeds of various predetermined length^28^ by a proper design of magnetic substrate symmetry and the use of proper external field sequence. Thus at the cost of lower transport velocity, which is required for the motion to be adiabatic and thus well controllable, the system offers high precision of both synthesis and consecutive transport^28^.

In our present paper, we introduce a ferrimagnetic Tb/Co^29^ PMA ML, which has been patterned using focused ion beam technique (FIB), as a prospective system for magnetophoretic applications.

## Results and discussion

As previously shown, the magnetic properties of Tb/Co ferrimagnetic MLs can be significantly changed using methods such as ion bombardment^29–31^ and plasma oxidation^32^. In these cases, the treatment of the films with energetic ions leads to a preferential oxidation of Tb relative to Co, and reduces the contribution of Tb to the effective magnetization. Thus, for high enough ion dose *D*, the direction of effective magnetization in the bombarded areas might reverse from being parallel to the magnetization of the Tb sublattice (as is the case for the $\textrm{Tb}+$ as-deposited regions of the film^33,34^), to having the magnetization direction of the Co sublattice ($\textrm{Co}+$). The effective magnetization ($M_{\textrm{eff}}$) approaches zero at the so called compensation composition value, $c_{\textrm{Tb}}^{\textrm{comp}}$. Note that an easy axis of magnetic anisotropy is not affected by the bombardment for the range of *D* used and the coercive fields are inversely proportional to $M_{\textrm{eff}}$ ^30,33,34^. The $\theta _{Kerr}$(*H*) dependencies were determined by integrating the gray values from differential images obtained from Kerr effect microscope (in polar configuration)^35^. The background image was taken at saturation (maximum applied field in the negative direction). The sign of the Kerr signal is determined predominantly by the Co sublattice^36–39^. Therefore, when the film is $\textrm{Tb}+$, the change in contrast in the differential image is opposite to the change in contrast when the film is $\textrm{Co}+$.

**Figure 1:**
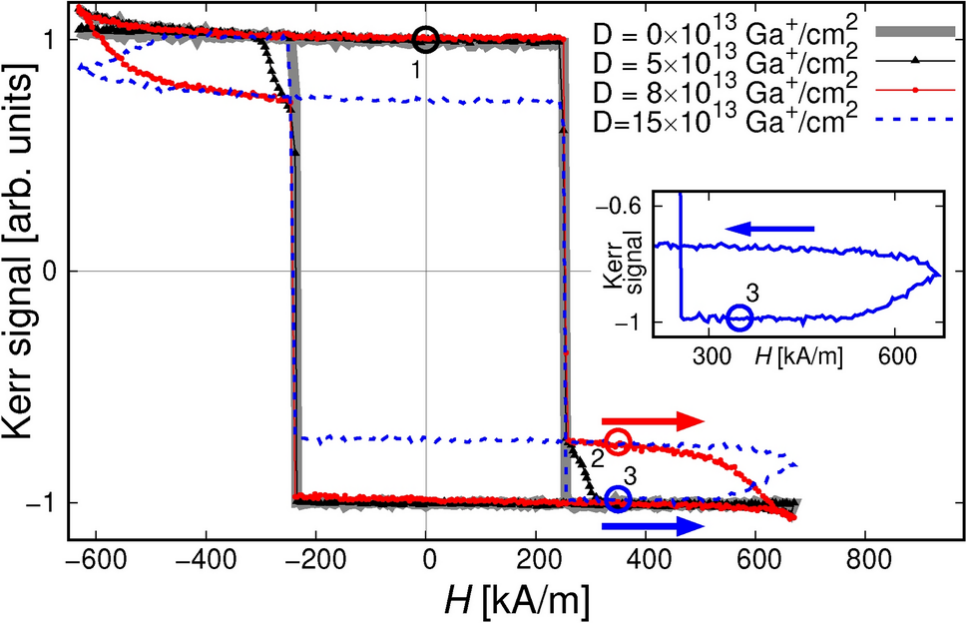
Magnetization reversal of the as-deposited Tb/Co ML and that of the bombarded arrays of 2 $\mu$m squares calculated from Kerr effect microscope images. Note that in our P-MOKE setup, the Co subsystem magnetic moments determine the magneto-optical signal. In the as-deposited state, we observe just a square loop with the coercive field of some 240 kA/m. Thick circles relate to the bombarded structures magnetic configurations of which are retained after the external field is switched off. The circle no. 1 corresponds to Fig. 2a, the circle no. 2 corresponds to either of the configurations shown in Fig. 2b (*D* = 8$\times$10$^{13}$ Ga$^+$/cm$^2$) and  2d (*D* = 15$\times$10$^{13}$ Ga$^+$/cm$^2$), and the circle no. 3 corresponds to Fig. 2c. For better visibility, the inset shows only the dependence for *D* = 15$\times$10$^{13}$ Ga$^+$/cm$^2$.

**Figure 2:**
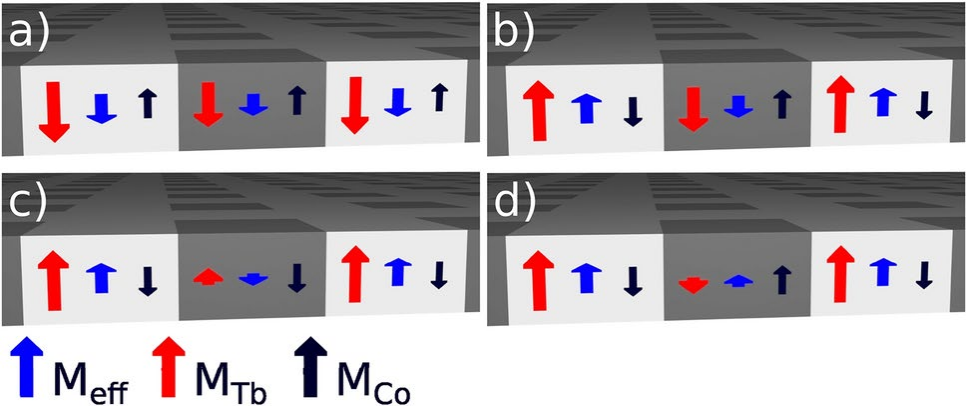
Schematic cross section of the magnetic structure of the Co/Tb bombarded MLs. For smaller doses of Ga$^+$ ions the squares can still show Tb moment domination [(a) and (b)]. Depending on the relative orientation of the moments of the squares and matrix, we can observe the rotational-type domain walls [(b) and (d)] or the abrupt transition between oppositely magnetized domains without associated gradual spatial rotation of spins within the transition region [(a) and (c)] [compare Fig. S1 in SI].

Exemplary $\theta _{Kerr}$(*H*) dependencies of the bombarded structures are shown in Fig. 1. For the pristine sample, the reversal is completed in one step. In the patterned region (see Section 4.1) however, the bombarded squares’ magnetization switches at higher fields, resulting in two steps in the reversal process. For the highest *D* ($\textrm{Co}+$ squares, dashed line), the signal reaches absolute values higher than those at the saturation. At the negative saturation (i.e., at $H\approx$ -600 kA/m) the effective magnetic moments of the squares and the matrix point in the same direction (compare Fig. 2d) but the moments of the Co subsystem are oppositely oriented, resulting in an intermediate signal. At $H\approx$ 240 kA/m, as the matrix magnetization reverses, the Co moments in the whole structure are parallel (Fig. 2c) and the observed signal is highest. Based on the relative orientations of the blue arrows ($M_{\textrm{eff}}$) in the panels of Fig. 2, from here on we will call the arrangements of panels (a) and (d) the parallel, P, configuration; and that of other two, AP (antiparallel). Note that the external magnetic fields we use in the magnetophoresis, see below, do not exceed 4 kA/m; this means that the magnetic structure of the (Tb-1.05 nm/Co-0.66 nm)$_6$ ML is not influenced by them.

As a side note we remark that irrespective of the mutual orientation of the effective magnetization of the squares and the matrix we expect magnetic phase boundaries^29^ (see Fig. S1 of Supplementary Information (SI)). This fact, however, seems not to be directly relevant for applications in magnetophoresis using Tb/Co ML, because the beads float above the substrate at heights which are dozens of times greater than an estimated wall width (approx. 20 nm^29^) and their volume, relevant for magnetostatic forces, is on average much farther.

The magnetostatic force acting on the superparamagnetic particles depends on the gradient of the square of the magnetic field, $\vec {H}$ ^40^:

1 $$\begin{aligned} \vec {F}_m = \frac{1}{2}\mu _0 \chi _{\textrm{eff}} V\nabla (\vec {H}^2), \end{aligned}$$

where $\chi _{\textrm{eff}}$ is the effective magnetic susceptibility (the difference between the bead’s and the fluid’s susceptibilities with $\chi _\mathrm {{\scriptscriptstyle H_20}} \approx$ -9$\times ~10^{-6}$ ^41^) and *V* is the bead’s magnetic volume^42,43^. We assume that $\chi _{\textrm{eff}}$ is constant for the weak magnetic fields of our experiment. In the case of Dynabeads^®^ M-270, *V* is virtually their entire volume as can be inferred from the manufacturer’s description “*magnetic material precipitated in pores evenly distributed throughout the particles*”^44^. The average effective magnetic anisotropy energy of the precipitates in M-270 beads is of the order of 1$\times$10$^{\mathrm {-19}}$ J and the attempt frequencies are of the order of 10$^{\textrm{17}}$ Hz (Table 2.3 in Ref.^45^); these values, taking into account the uncertainties and using the usual exponent formula for a magnetization Néel relaxation time (equation (2) in reference^46^), give the $\tau _{\textrm{N}}$ values from 1$\times$10$^{\mathrm {-4}}$ s down to 1$\times$10$^{\mathrm {-13}}$ s. Since the frequency of field change, $f_{\textrm{rot}}$, in our experiment do not exceed 12 Hz we may assume that the magnetization of the SPBs instantaneously follows the direction of the external field.

The SPBs in suspension are attracted to regions of high $H^2$ (Eq. 1), so that above thin films they preferentially settle wherever there is a change of effective magnetization. This is readily seen in our Tb/Co arrays: the beads decorate the borders between the matrix and the FIB-ed areas (see Fig. S2 in SI). We observe the same behaviour for all Ga$^+$ doses used and for squares with sides $a\ge$ 10 $\mu$m.

A requirement for use in magnetophoretic applications is that the substrate provide high gradients of field and the resultant energy landscape, *E*($\textbf{r}$). It is also necessary to be able to change the forces acting on the SPBs so that they can traverse the substrate in a controlled manner. This can be achieved by applying an external, gradient-free, magnetic field which temporarily changes the magnetostatic forces. A proper sequence of the external field directions can lead to SPB displacement of one lattice spacing per period of the rotating external field $H_{\textrm{ext}}$. The root cause of the movement is that changing the external field shifts the energy minima. This means that a SPB placed in the location of a previous minimum will experience a force that drives it towards the energy minimum’s new location. Since the underlying physics has already been worked out in numerous papers (e.g. ^19,20,47,48^), we consign the specifics of our arrays to the SI.

**Figure 3:**
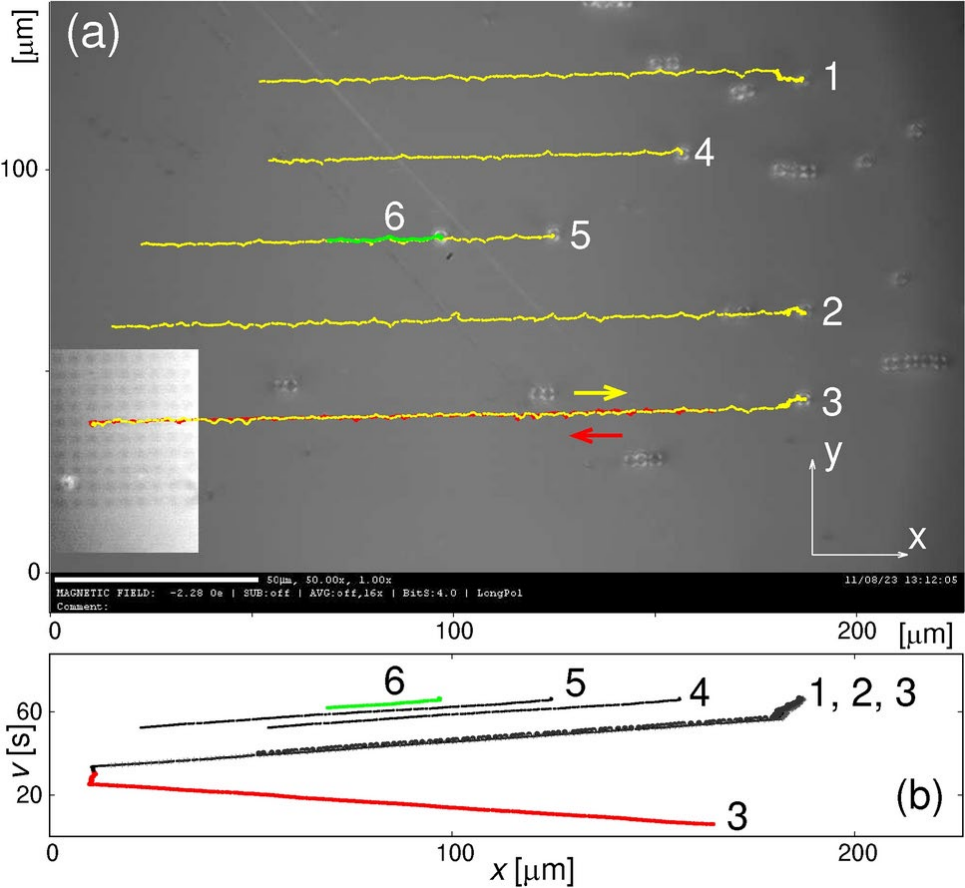
(a) Selected, meaning not representative (see the text), traces of the SPB’s trajectories superimposed on the frame from the corresponding movie (see the movie no. 2 in SI). All traces end to the right (the individual tracked beads are visible there). During the experiment the field of $\sqrt{2}\times$1590 A/m strength was switched (see Fig. 7) at 2 Hz frequency. The traces are colored for better visibility. The red part of one trace corresponds to the movement in the opposite direction [compare (b)]. The bright rectangle in the lower-left corner of the image accentuates the underlying magnetic structure with 2 $\mu$m wide squares; it was obtained by increasing the contrast of the average of 9 frames of the movie centered at the one shown in the rest of the image. (b) The *x*-coordinates of the beads/traces as a function of time showing that they all move with the same average velocity; note that three traces, ending at *x*$\approx$180 $\mu$m, correspond to in unison motion of the beads.

The trajectories of several beads are shown in Fig. 3. They correspond to simultaneous movement of several SPBs with the external field switching at a frequency, $f_{\textrm{rot}}$ = 2 Hz. The highlighted beads move in unison and three of them with approximately the same *x*-coordinate (see movie no. 2 in SI). The lateral deviations of trajectories from the straight line result from the details of the magnetostatic energy landscape of SPBs for a square array (see Fig. S6 in SI): although in the absence of the $H_{\textrm{ext}}$ (i.e., at the start of the experiment) the minima are at the centers of the FIB-ed squares, the switching of $H_{\textrm{ext}}$ may move the minima to outside of the squares. Therefore the bead has a tendency to temporarily deviate off the straight line instead of following the projection of the red curve on the sample’s plane (it is best visible in a trajectory in Fig. S3 from SI). We observe magnetophoresis for all Ga$^+$ doses used and for both P and AP configurations (see Fig. 2, and below). Although we analyze only the behaviour of single SPBs, aggregates of several beads can also be transported (compare Ref.^49^ and see the movie no. 2 in SI). Sometimes this aggregate motion takes place at frequencies for which single beads can no longer track the shifting potential minima.

**Figure 4:**
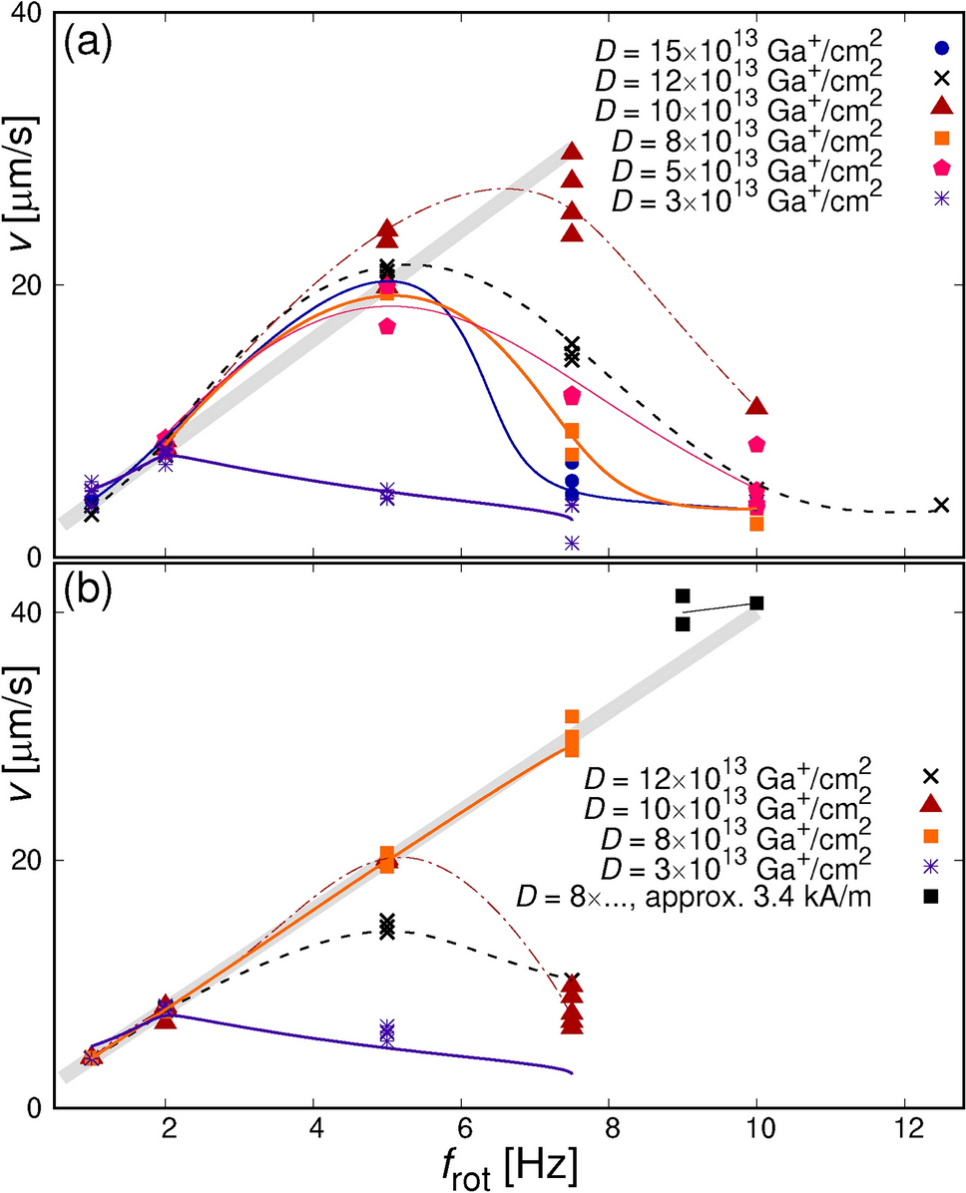
Dependencies of average velocity of the SPBs on the switching frequency of the magnetic field of $\sqrt{2}\times$1590 A/m, for parallel (a) and antiparallel (b) nominal orientations (see the text) of the effective magnetization in the matrix and in the bombarded squares (see Fig. 2). The squares were 2 $\mu$m wide. The dependencies for bombardments with various doses of Ga$^+$ ions are shown. The faint thick straight lines correspond to the “phase-locked” regime (see the text) with $v=4\cdot f_{\textrm{rot}}$ [$\mu$m/s]; the other lines are just for better viewing. The eye-guiding line for *D* = 3$\times$10$^{13}$ Ga$^+$/cm$^2$ is the same in (a) and (b). Note that in (b) black squares correspond to higher applied field. For the discussion of errors see Section 4.

The dependencies of an average velocity *v* of the SPBs on $f_{\textrm{rot}}$ (Fig. 4) show a characteristic drop above some critical frequency $f_{\textrm{c}}$, which is roughly in the 5–7.5 Hz range (for the smallest *D*, the $v(f_{\textrm{rot}})$ dependence deviates from linearity significantly earlier). Above $f_{\textrm{c}}$, and within a reasonable range above it ($v(f\rightarrow \infty )\rightarrow 0$ ^47^), the velocities of the individual SPBs, for a given *D* and orientation (P or AP), have significantly greater scatter than below. This is due to the inevitable spread of magnetic and hydrodynamic properties of the individual beads and is virtually irrelevant for prospective applications where $f_{\textrm{rot}}<f_{\textrm{c}}$ (compare with topologically protected transport^26^). The existence of $f_{\textrm{c}}$ is caused^48^ by the viscosity of the liquid in which SPBs move, which in our experiments is relatively viscous water. When the positions of minima shift too frequently, the magnetic particle does not reach them and instead oscillates at an intermediate position. Up to $f_{\textrm{c}}$ the *v* increases approximately linearly with $f_{\textrm{rot}}$ and it ought to follow a $v=2 a f_{\textrm{rot}}$ dependence, as SPBs should move by one lattice spacing per period $T_{\textrm{rot}}=1/{f_{\textrm{rot}}}$ (this is the so called “phase-locked” regime^47^). It is the case as visualized with thick faint straight lines in Fig. 4. (Instantaneous velocities vary significantly within $T_{\textrm{rot}}$ though — see Fig. S3 of SI or Ref.^48^.) Since the only prerequisite for magnetophoresis is the existence of regions with the effective magnetization, $M^{\textrm{eff}}_{\textrm{sqr}}$, different than that of the matrix ($M^{\textrm{eff}}_{\textrm{mat}}$), we consistently observe magnetotransport for the two nominally distinct relative orientations of the effective magnetization of the matrix and the squares, P and AP.

The general dependence of $v(f_{\textrm{rot}})$ on *D* (see Fig. 4a), that is the increase of $f_{\textrm{c}}$, follows directly from the above mentioned changes of the $M^{\textrm{eff}}_{\textrm{sqr}}$ with *D*. The maximum attainable *v* values for lowest *D* are distinctly smaller than for higher doses because the smallest difference between $M^{\textrm{eff}}_{\textrm{sqr}}$ and $M^{\textrm{eff}}_{\textrm{mat}}$ produces the weakest magnetostatic fields above the thin film. In the AP configuration, for *D* = 3$\times$10$^{13}$ Ga$^+$/cm$^2$, one would expect highest *v* values then, which is evidently not the case — the velocities are again smallest. Our explanation is that the switching field of the squares for that small dose is virtually equal to that of the matrix (compare the curve for *D* = 5$\times$10$^{13}$ Ga$^+$/cm$^2$ in Fig. 1) and the whole array reverses simultaneously. It is nicely confirmed by the fact that, for *D* = 3$\times$10$^{13}$ Ga$^+$/cm$^2$, the discrepancies between $v(f_{\textrm{rot}})$ for the P and AP cases in Fig. 4 are within measurement error. We note, too, that the dependencies for *D* = 8$\times$10$^{13}$ Ga$^+$/cm$^2$ and *D* = 10$\times$10$^{13}$ Ga$^+$/cm$^2$ are, in a sense, conjugate: changing the configuration from P to AP (see the discussion of Fig. 1) swaps the $v(f_{\textrm{rot}},D)$ dependencies ($v(f_{\textrm{rot}},10\times 10^{13}~\textrm{Ga}^+/cm^2)_{AP}\sim v(f_{\textrm{rot}},8\times 10^{13}~\textrm{Ga}^+/cm^2)_{P}$, and similarly for the second pair of dependencies). Disregarding the details of the configuration of magnetic moments at boundaries of the squares, we know that the magnetic field acting on the beads is a monotonic (in the mathematical meaning) function of $|M^{\textrm{eff}}_{\textrm{mat}}-M^{\textrm{eff}}_{\textrm{sqr}}|$. Thus, this behaviour indicates that for both of these doses, the effective magnetizations of the squares are close in magnitude but the magnetic moments of the Co subsystem are opposite to the net magnetization for the smaller *D* and parallel for the higher *D*.

Applying higher external fields, by increasing the force acting on SPBs, allows $f_{\textrm{c}}$ to shift to higher values, and thus to achieve higher *v* accessible for applications. In our experiments, using the highest available strength of the switching field, the maximum velocities were above *v* = 40 $\mu$m/s [black rectangles in Fig. 4b].

**Figure 5:**
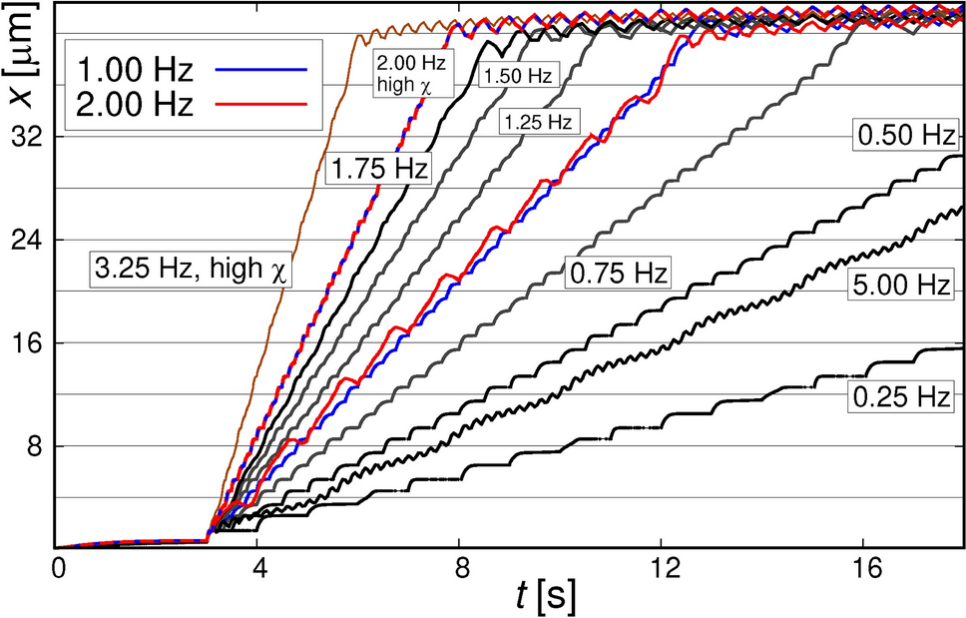
Simulated trajectories (see the Section 4.3) of the beads (*x*-coordinates), moving over the array of 2 $\mu$m cubes, for various frequencies $f_{\textrm{rot}}$ of the field switching. The beads reaching the outer edge of the magnetic array (at *x* = 40 $\mu$m) oscillate there as the field rotates. The horizontal lines spacing corresponds to the spatial period of the structure. The magnetization of the squares was set to 0.5424$\times$10$^{6}$ A/m. The brownish and blue-red curves correspond to the SPB with susceptibility of 0.35^46^; for the other dependencies $\chi _{\textrm{eff}}$ = 0.22^50^.

**Figure 6:**
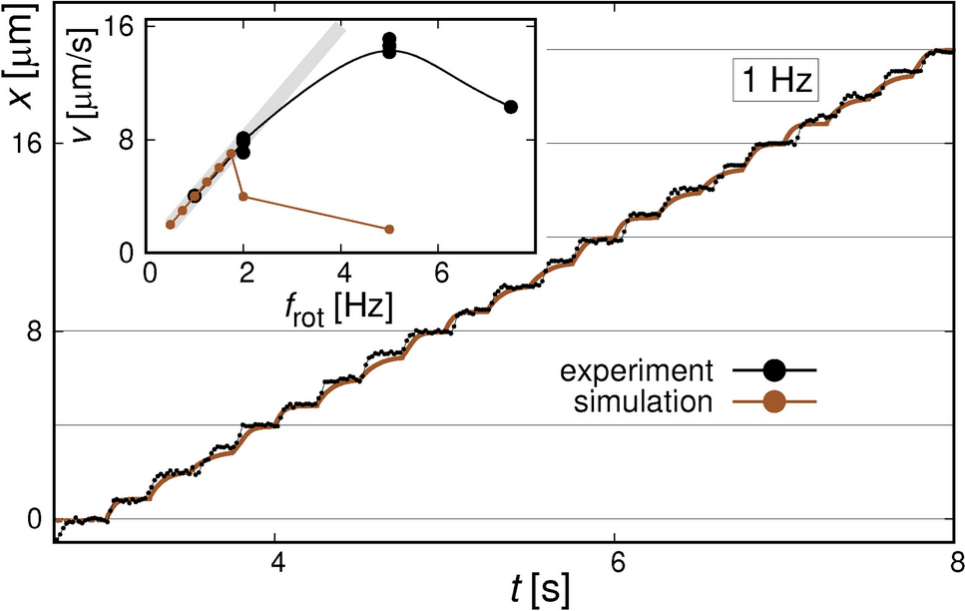
Simulated and measured *x*-trajectories of the bead moving over the array of 2 $\mu$m cubes with the external field switching at $f_{\textrm{rot}}$ = 1 Hz. The inset show the dependencies of the SPB velocities, averaged over several periods of the field variations, on $f_{\textrm{rot}}$. The experimental curve corresponds to the curve for *D* = 12$\times$10$^{13}$ Ga$^+$/cm$^2$ from Fig. 4b and the simulated one is calculated from the data of Fig. 5.

The *v* values we report here depend on both the external gradient-free field and the magnetic field of the substrate, $\vec {H}_{\textrm{sub}}$, which determine the relevant gradient (Eq. 1) according to the formula: $\nabla (\vec {H}_{\textrm{sub}}+\vec {H}_{\textrm{ext}})^2=\nabla (\vec {H}_{\textrm{sub}}^2)+2\nabla (\vec {H}_{\textrm{sub}}\cdot \vec {H}_{\textrm{ext}})$. We have previously shown^34^ that the magnetic properties of the Tb/Co ML are preserved up to at least 15 repetitions of the basic bilayer. The same magnetophoretic velocities can thus be obtained with weaker $\vec {H}_{\textrm{ext}}$ by using thicker films (with a penetration depth of ions used being a limiting factor) or can be increased using stronger $\vec {H}_{\textrm{ext}}$.

One of the crucial parameters determining the magnetostatic fields produced by the array is the saturation magnetization, $M_{\textrm{S}}$, of the pristine and bombarded areas of the sample. However, we do not have the possibility to determine these values independently; instead we estimate them using the coercivity as a measure of relevant changes. Ion bombardment strongly modifies the anisotropies and interfaces in magnetic multilayers; however, it has been shown that the changes in magnetic properties of RE/TM multilayers are mainly due to preferential oxidation of Tb^31,51^. The amount of oxidized Tb can be estimated by comparing the change in coercivity versus dose with the coercivity measured with a multilayer composed of a wedge of the Tb sublayers. Thus, assuming that the bombarded region is equivalent to a multilayer with a reduced Tb thickness and using Eq. 1 from Ref.^51^ we obtain estimates of the $M_{\textrm{S}}$ values (see the Table 1 in SI). For example, to calculate the magnetic field produced by the array of the squares bombarded with 12$\times$10$^{13}$ Ga$^+$/cm$^2$ dose, and the oppositely magnetized matrix (AP configuration), we use the $M_{\textrm{S}}=0.494-(-0.048)=0.542$ MA/m for the magnetization of the squares (where 0.494 MA/m is $M_{\textrm{S}}$ of a pristine matrix) with the $M_{\textrm{S}}$ of infinite matrix set to zero.

We have made some attempts at simulating the experimental trajectories taking into account magnetic, viscous, electrostatic, and some other forces present in our system (see section  4.3). The saturation magnetization of the bombarded squares, needed to calculate the magnetic fields, was estimated as described above. The basic results are shown in Figs. 5 and  6, and in Fig. S7 of the SI. The calculated *x*(*t*) dependencies show the expected behaviour with the beads moving with field rotation to consecutive energy minima. Up to the frequency of 1.75 Hz the beads move in “phase-locked” regime, i.e., their velocity is proportional to the $f_{\textrm{rot}}$. At higher frequencies the magnetic forces exerted on the beads are not sufficient for them to be able to reach the position of the next minimum before the consecutive field direction change takes place. Comparing the experimental trajectories with modeled ones (Fig. 6) one can see that at low frequencies the agreement is satisfactory. However, the mobility of the SPBs is underestimated in the simulation: in the experimental curve the beads quickly approach the equilibrium *x* positions corresponding to a momentary field orientation while in the simulation they only reach that position just before the field reorients. Consequently, the modelled dependence of SPBs average velocity on $f_{\textrm{rot}}$ (see the inset of Fig. 6) shows critical frequency of some 1.75 Hz, while in the experiment this frequency seems to be close to 4 Hz (we do not have enough experimental trajectories to determine it more precisely). We believe the difference to be caused mainly by the underestimation of the magnetic susceptibility $\chi _{eff}$ of the beads. The SPBs have a tendency to float some distance above the PMMA spacer because of double-layer electrostatic repulsion^52^ and this influences the magnetostatic forces. However, test simulations with varying the distance of the bead to the substrate [by slightly varying the Debye length (see section  4.3)] show that this only weakly influences the trajectories. In a model trajectory of Fig. 6 the beads travel at a distance 0.8–1.3 $\mu$m from the substrate; the equilibrium distance itself depends on the direction of the external field. On the other hand, literature data points to high spread of the $\chi _{\textrm{eff}}$ values even for the specific SPB type we use ^46,50^. Taking for example the $\chi _{\textrm{eff}}$=0.35^46^ instead of 0.22^50^, determined from slightly wider field range, brings the motion of the bead back to “phase-locked” regime (compare red and red-blue curves in Fig. 5) and significantly increases the $f_{\textrm{c}}$ - see brown curve there. For these reasons, we attribute the disagreement between simulated and experimental $f_{\textrm{c}}$ values to the dispersion of the $\chi _{\textrm{eff}}$ and disregard the distance effects.

Although we did not perform the experiments aiming at optimizing the geometry of the magnetic substrate for a given SPB’s diameter, as it is well described in literature^19^, we performed some simulations for varying *a* value. The results are presented in Fig. S7 of SI. The main conclusions from this set of calculations is that for SPB’s diameter of 2.8 $\mu$m the highest transport velocities are expected for the square’s edges of some 4–5 $\mu$m. However, we should note that the calculation was performed for specific values of parameters determining the distance of the SPB from the substrate (DLVO interactions, etc. ^52^) which influences the magnetic forces acting on the beads and thus the values of the $f_{\textrm{c}}$.

## Conclusions

In this paper we have shown potential applicability of structured Tb/Co multilayers for magnetophoretic transport of superparamagnetic, micrometer sized beads in an aqueous environment. We used a focused beam of Ga$^+$ ions to locally change magnetization of the film and to create the magnetostatic energy landscape that enables controllable transport of the beads with velocities of 40 $\mu$m/s using the external fields of approx. 3370 A/m (4.2 mT).

## Methods

### Sample preparation and the magnetophoresis experiments

The Ti-4 nm/Au-30 nm/(Tb-1.05 nm/Co-0.66 nm)$_6$/Au-5 nm ML was prepared on a naturally oxidized Si(100) substrate via magnetron sputtering in an Ar atmosphere (1.56$\times$10$^{-3}$ mbar). The base pressure in the chamber was 1.42$\times$10$^{-8}$ mbar. The deposition rates were calibrated with pilot samples whose thickness was determined using a Bruker DektakXT stylus profiler. Deposition rates for Au, Ti, Tb, and Co were 0.111, 0.043, 0.035, 0.037 nm/s, respectively. The sample fabrication method is given in more detail in References^29,34^.

**Figure 7:**
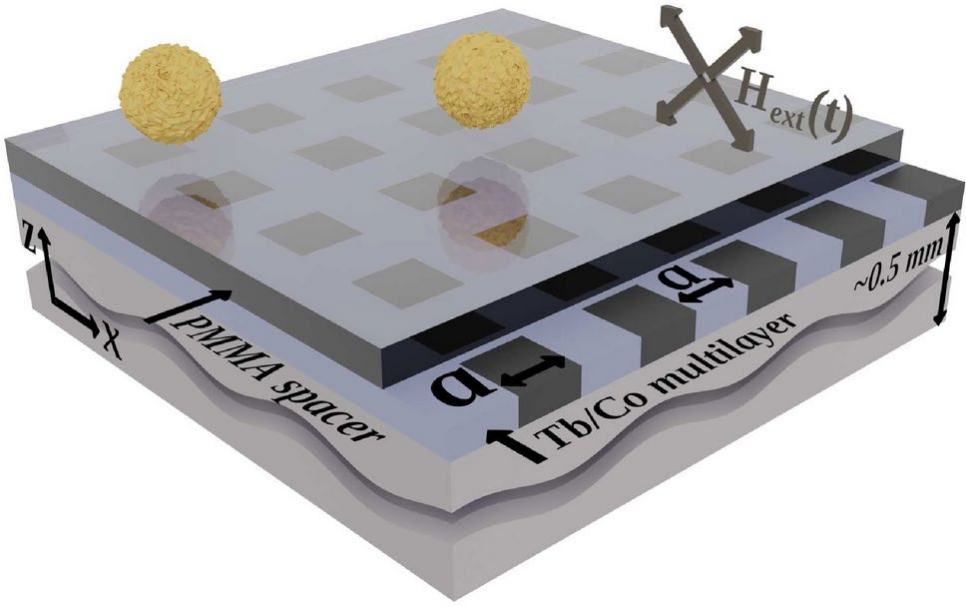
Schematic of the experimental setup. The superparamagnetic 2.8 $\mu$m diameter beads (yellow spheres) float in water above a PMMA spacer which covers the magnetic structure. The approx. 90 nm thick spacer acts as a protective layer for the reactive Tb/Co thin film deposited on naturally oxidized Si substrate. The Tb/Co layer is patterned with focused ion beam into several arrays of squares (shown in darker gray) with magnetic properties different from that of the matrix. The distance between neighboring squares is the same as the length of their edges, *a*. The external magnetic field switches between the four directions within the *xz*-plane causing the beads to move. The thicknesses of the layers are not to scale.

A series of 2D periodic lattices were patterned using a 30 keV Ga$^+$ FIB (FEI Helios Nanolab 660 workstation). The lattices covered an area of 200$\times$200 $\mu$m$^2$ each, and consisted of bombarded squares with edge lengths and edge-to-edge separation distances *a* = 1, 2, 10, and 50 $\mu$m (Fig. 7). Each structure was patterned with approximately uniform ion doses *D* = 3, 5, 8, 10, 12, or 15$\times$10$^{13}$ Ga$^+$/cm$^2$. In the as-deposited state, the effective magnetization of the sample is dominated by the Tb sublattice^30,34^.

The magnetic array was subsequently covered with a nominally 90 nm thick polymeric PMMA protective layer (All Resist^53^, AR-P671.02) using spin-coating at 4000 rpm. In contrast to, for example, Co/Au systems^24^ this step is necessary to perform magnetophoretic experiments because Tb is highly reactive with water^54^.

The magnetic properties were measured with a polar magneto-optical Kerr effect P-MOKE microscope (evico magnetics GmbH).

The magnetophoretic experiments were performed at room temperature, in an aqueous environment (laboratory grade (i.e. osmosis, plus double demineralization) water from a purifier^55^); the sample was placed in a printed vessel (made using PLA type filament) and covered with approximately 0.3 mm of suspension of superparamagnetic Dynabeads^®^ M-270 beads^56^. The original suspension was diluted 1:1000 to obtain a final concentration of approx. 2$\times$10$^6$ beads/mL. The experiments were recorded with our microscope equipped with Hamamatsu Photonics Orca-spark model C11449-36U camera through an infinity corrected LD EC Epiplan-NEOFLUAR 50$\times$/0.55 DIC objective lens with 25 mm extension (needed to reach the center of the field coils). The movies (960 pixels $\times$ 642 pixels) were recorded with frame rates of 32-55 fps (depending on the image brightness). The differences in visibility of squares between images are due to the polarizer used in our microscope [compare Figs. S2 and S3 in SI].

The magnetic field, $H_{\textrm{ext}}$, in the experiment was obtained from two sets of Helmholtz type coils, in a set-up similar to that of Ref.^24^. The coils were powered with KEPCO BOP 100-4DL bipolar power supplies. Both coil sets produce a field of approx. 1600 (A/m)/A (2 mT/A). The input signal of the supply of the bigger coils (providing *x* component of the field) was digitally ramped to minimize overshoot and ringing due to their higher self-inductance of approx. 4.5 mH; the resulting switching time was 20 ms. For small coils the switching time is less than 0.2 ms with small and short ringing.

### Velocimetry

Traces of the SPBs’ in-plane (*xy*) trajectories (referred to as trajectories for brevity) were obtained from recordings analysed with the image tracking Video Spot Tracker software^57^. The tracking kernel (“image”) was set to either 5 or 6 pixels diameters. Note, however, that despite this kernel outperforming the others, it often loses track of the SPBs. These lapses are consequences of variations of focus and background contrast during the experiment. For example, the fluctuation in contrast affects (among others) the visibility of the squares. In some cases, the lapses are only a few frames long and the continuity of the individual SPB’s trajectory is restored. Consequently, roughly 20% of the recorded traces are usable. There are numerous sources of errors inherent in such types of image-based velocimetry^58^. We consider the main error sources to be: (i) uncertainty in resolving the trajectories (due, e.g., to the initial placement of the tracker), (ii) uncertainty in the fitting time range at the available frame rates (these intervals are usually not multiples of the $H_{\textrm{ext}}$ period), and (iii) uncertainties in the frame rate of the recording. To estimate the error, we take the sample standard deviation of all SPB velocities at external field switching frequency $f_{\textrm{rot}}=$ 2 Hz (a total of 47 values, all shown in Fig. 4). We are confident that at this frequency the SPBs are “phase-locked” (see Section III) and should be moving with a 8 $\mu$m/s velocity. The standard deviation is slightly less than 5% of the mean. For clarity, we do not show error bars in Fig. 4. To average the recording frames (see the bright rectangle of Fig. 3) we used ImageJ software^59^.

### Modelling

To model the transport of the SPBs over our structure we used an approach analogous to that described in Ref.^48^. The simulations included the magnetic forces exerted by the array of magnetized cubes, viscous forces due to the aqueous SPB environment, electrostatic forces (double-layer repulsion) between surfaces of the beads and the substrate^52,60^, van der Waals forces^61^, as well as buoyancy, and gravity. To calculate the magnetostatic force acting on the SPBs we used formulas of Kuleznev et al.^62^ for magnetic field of uniformly magnetized cubes. In the calculation we divide the bead’s volume into 64 cells (with 4 divisions along the radius, the azimuthal angle, and polar angle respectively) and for each of them we calculate the force according to Eq. 1; note that the division into 8$\times$8$\times$8 cells changes the trajectories only insignificantly as the beads float relatively high above the substrate (in the simulation). We assume the magnetic susceptibility of 0.22^50^. To calculate the electrostatic forces between the SPBs and the substrate we use a analytic derivative of an equation 1b from Ref.^60^ (Hogg-Healy- Fuerstenau formula). $\zeta$ potential value for PMMA resist in water was taken to be 24 mV (Fig. 4a in Ref.^63^), and that of carboxylic acid functionalized M-270 beads -36 mV^64,65^. The Debye length $\lambda _{\textrm{D}}$ of our water was assumed, somewhat arbitrarily to be 200 nm, as we can expect slight deviations of pH due to the presence of impurities on the substrate or, for example, incorporation of atmospheric CO$_{\textrm{2}}$ ($\lambda _{\textrm{D}}$ of the deionized water can be as high as 1$\mu$m^61^ and usually” is only a few 100 nm due to ionic impurities or a pH different from pH 7.”^66^). To calculate van der Waals interactions, which are in fact negligible relative to electrostatic interactions for bead-to-surface distances present in our system, we use the analytic derivative of an equation 4.8 from Ref.^61^. As we do not have the Hamaker constant, A$_{\mathrm {C-P}}$, for interaction of carboxylic acid functionalized surface with PMMA surface, we use the geometric average (Eq. 4.13 in^61^) of Hamaker constants for PMMA surfaces interacting in water A$_{\mathrm {P-P}}$ = 1$\times$10$^{\mathrm {-20}}$ J ^67^ and A$_{\mathrm {C-C}}$=1.2$\times$10$^{\mathrm {-19}}$^68^ for carboxyl group terminated surfaces. The viscous force acting on the SPBs was calculated from Stokes’s law with a correcting drag coefficient which depends on a SPB’s surface to the substrate distance - see Eq. 6 in Ref.^69^. The gravitational force was calculated with a beads density of 1.6 g/cm$^{\textrm{3}}$^44^. The magnetization of the bombarded areas was estimated from the switching fields (see Sect. 2). The relative permeability of water was taken as 80.4, and its viscosity set to 1 mPa$\cdot$s.

To model the trajectory of the SPBs, *r*(*x*(*t*), *y*(*t*), *z*(*t*)), we utilized the fact that the bead’s motion in the viscous fluid, for the low field switching frequencies we use, can be assumed to be taking place without an acceleration: the bead attains its steady state velocity ($v_{\textrm{SPB}}$) within several microseconds (sic!) from the application of the force (Eq. 5 in Ref.^48^). The $v_{\textrm{SPB}}$ is thus the function of the bead’s position and the time, but only through the varying direction of the external field. We simulate the trajectories over the array of 10$\times$10 cubes of 10 nm thickness (corresponding to the thickness of the magnetic (Tb-1.05 nm/Co-0.66 nm)$_6$ multilayer). Initially the bead is placed at the height of 2.9 $\mu$m over the PMMA spacer, at *x* = 0.1 $\mu$m, and at *y* corresponding to the symmetry plane of the structure parallel to the *x*-axis (i.e., the starting *y* position depends on the width of the cubes). We then allow the bead to move under the influence of the internal forces, and at t = 3 s we switch on the varying field of the strength equal to that used in the experiments. For advancing the SPBs position we use an adaptive algorithm^70,71^ with time steps down to 0.5 ms. Note that only a single bead is included in our simulations.

## Electronic supplementary material

Below is the link to the electronic supplementary material.

Supplementary Information 1.

Supplementary Information 2.

Supplementary Information 3.

## Data Availability

The datasets generated during and/or analysed during the current study are available in the zenodo.org repository, https://zenodo.org/doi/10.5281/zenodo.10817106.
