# Supplementary material for: Ferrimagnetic Tb/Co multilayers patterned by ion bombardment as substrates for magnetophoresis
Source: Sci Rep. 2024 Oct 10;14:23771. doi: 10.1038/s41598-024-73203-3 (PMC11467345; doi:10.1038/s41598-024-73203-3)
Supplement: Supplementary file 1 — Supplementary Information 1. [file 41598_2024_73203_MOESM1_ESM.pdf]

Ferrimagnetic Tb/Co multilayers patterned by ion bombardment as substrates for magnetophoresis
Supplementary Information

Maciej Urbaniak,[*] Daniel Kiphart,[†] Michał Matczak,[‡] Feliks Stobiecki,[§] Gabriel David Chaves-O'Flynn,[¶] and Piotr Kuświk[**]
Institute of Molecular Physics,
Polish Academy of Sciences,
Mariana Smoluchowskiego 17 60-179 Poznań, Poland

In the main text of the paper we mentioned peculiarities of the local magnetic configuration in the boundaries between the as-deposited regions of Tb/Co (Ti-4 nm/Au-30 nm/(Tb-1.05 nm/Co-0.66 nm)$_6$/Au-5 nm) ferrimagnetic multilayer (ML) and the areas bombarded with Ga^+ ions. A schematic (Fig. S1), approximately corresponding to our previous micromagnetic simulations of the boundary regions [1], shows that when the effective magnetizations ($M_{\rm eff}$) of Tb dominated (i.e., with most of the magnetic moment originating from Tb atoms) and Co dominated (bombarded) areas are parallel, the spins of both subsystems rotate in space like in a typical ferromagnet. The transition region is some 20 nm wide [1]. When, however, the magnetization of the squares is opposite to that of the matrix, there is no rotation and the orientation change is abrupt — there are no rotational-type domain walls in the system. As already mentioned in the main text, and as demonstrated by dependencies of Fig. S4 and Fig. S5, the averaging effect of the large diameter of the superparamagnetic beads (SPBs) makes the details of the magnetostatic fields in the direct vicinity of the domain walls irrelevant for our experiments, although they are important for the stability (i.e., switching fields, etc.) of the magnetization textures [1].

We observe (Fig. S2) a sedimentation of the SPBs in direct proximity of the boundary between ion bombarded and pristine area irrespective of either the dose of Ga^+ ions used or the relative orientation of the $M_{\rm eff}$ of the matrix and the squares (P or AP configuration—see Fig. 3 in the main text). This alone indicates the presence of the energy landscape which is potentially usable in magnetophoresis. Note that in contrast to this behaviour, the same SPBs are attracted to the centers of the squares if their edges are comparable in length, a, to the diameter of the beads (Fig. S5, the curve for zero applied field).

The SPB trajectories reveal the characteristic features only when observed at sufficiently low field switching frequency, $f_{\rm rot}$ (i.e., compatible with recording frame rate). The movement of the bead shown in Fig. S3 is in the "phase-locked" regime [2]: the SPB moves by one lattice spacing of the magnetic structure per every period of the switching field cycle, $T_{\rm rot} = 1/f_{\rm rot} = 8$ s. For a given direction of the external field, $H_{\rm ext}$, there is a definite energy minimum position for the bead. Closer inspection of the black curve, say in the 614 to 622 s range, shows distinct plateaus on the $x(t)$ curve. Each of them corresponds to different energy minima, or more precisely to the lines containing those minima (see discussion of Fig. S6). Note that relative positions of the minima depend on the ratio of the SPB diameter to the lattice constant ($2a$, where a is a square size — compare Refs. [3, 4]); here the minima are very close to the edges of the squares as opposed to the case of $a = 2$ μm (compare Fig. S5).

As already mentioned in the main text of the paper, the magnetostatic force acting on the superparamagnetic particles depends on the gradient of the magnetic field strength squared, $\nabla(\vec{H}^2)$ [5]:

$$\vec{F}_m = \frac{1}{2}\mu_0\chi_{\rm eff}V\nabla(\vec{H}^2), \tag{1}$$

where χ is the effective magnetic susceptibility that we assumed to be constant for the weak magnetic fields acting on the beads in our experiment, and V is the bead's magnetic volume. Fig. S4 shows the xz map of the $\nabla(\vec{H}^2)$ over the structure corresponding to the investigated Tb/Co patterned arrays for certain $H_{\rm ext}$ direction. (For calculations we use field formulas from [6].) The x component of the gradient decreases steeply with h and is significantly higher in the vicinity of the magnetization discontinuities than in other areas. The force acting on the SPB is proportional to an integral of the $\nabla(\vec{H}^2)$ over its volume. To understand what causes the movement of SPBs, it is easier to use the energy landscape visualization instead. Fig. S5 shows an exemplary dependence of an integral of H^2 on the x position

[*] urbaniak@ifmpan.poznan.pl
[†] daniel.kiphart@ifmpan.poznan.pl
[‡] michal.matczak@ifmpan.poznan.pl
[§] feliks.stobiecki@ifmpan.poznan.pl
[¶] gabriel.chaves@ifmpan.poznan.pl
[**] kuswik@ifmpan.poznan.pl

of the bead's center. Note that the curves have only an illustrative character, and we are not interested in the exact height of the SPBs over the PMMA protective layer. The reason for this is that the height of the SPBs is necessary to predict the trajectories of SPBs (compare [3, 7, 8]) and this depends on electrostatic forces of the DLVO-type [9, 10].

We give now a short explanation of the magnetophoretic transport in our structures; note that the general principles were already described in numerous publications [2, 3, 7, 11]. If we imagine a SPB floating in a liquid over the array of the squares, it should sediment at $x = 21\ \mu m$ if there is no external field – dashed curve in Fig. S5; we neglect here the possibilities of the bead sticking to the surface, interacting with other particles etc. (We will use the following notation for the field direction: two signs relate to the orientation of components, respectively x and z, of the external field H_{ext}; the description "-+" denotes, for example the field of H_x = -1.59 kA/m, H_z = +1.59 kA/m.) When the "++" field is present the SPB gradually moves to the new, now significantly deeper, minimum. Switching the H_{ext} to "+-" orientation causes the position of previous minimum to be at a steep slope of a current $E(x)$ dependence and the bead moves towards the new minimum located at $x \approx 22.5\ \mu$m. Successive changes of H_{ext} move the particle farther to the right, to the minima localized over neighboring squares. If we reverse the sequence of changing H_{ext} directions the SPB moves to the left (compare Fig. 4 in the main text). Note however, that such a reversal does not in general, reverse the trajectory (i.e. for other magnetic substrate symmetries [12]).

Finally, we note that what looks like a minimum in Fig. S5 is in fact a saddle point (see the crossing of red and blue curves in Fig. S6) and in this field configuration the minima are in the xz-planes bisecting the areas between neighboring rows of squares parallel to x-axis. This explains, in our opinion, the repetitive deviations of trajectory from the straight line clearly visible on yellow curve in Fig. S3 and less so in trajectories of Fig. 4 of the main text.

The two movies, pr6904B10um.mov and pr6904B2um.mov, included in the Supporting Information, correspond to Fig. S3 of the present text and to the trajectories of Fig. 4 of the main text. The were obtained from the original files with ffmpeg converter, using command: *ffmpeg -i in.avi -c:v libx265 -crf 26 -preset fast -c:a aac out.mov* [13]. The single beads, in the second recording, that do not move with the external field are outside the array of squares. The movies were recorded with frame rates of 31 fps (pr6904B10um.mov) and 41 fps (the clock in the right corner shows the real time).

The simulations presented in the main text (Figs. 5 and 6 in the main text) and in Fig. S7 require the estimation of the saturation magnetization, M_{S}, of the areas of the sample bombarded with Ga^+ ions. The values obtaine using the method described in the main text are given in Table I. Note that the increase in M_{S} for a 15×10^{13} Ga^+/cm^2, relative to the next highest dose, is indicative of the change of the domination of the TbCo alloy to Co^+.

TABLE I. Estimated saturation magnetization of (Tb-1.05 nm/Co-0.66 nm)$_6$ MLs versus Ga^+ ion dose.

Dose [10^{13} Ga^+/cm^2]	M_{S} [MA/m]
0	0.494
3	0.483
5	0.462
8	0.286
10	0.178
12	0.048
15	0.092

The simulations of the trajectories of the SPBs presented in the main text (Figs. 5 and 6) were performed for the width of the squares of the array and the spacing between them corresponding to that used in our experiments (a = 2 μm). In Fig. S7 we show the set of simulated dependencies of the average velocity of the beads vs. f_{rot} for the arrays with scaled in-plane dimensions and with the thickness of the magnetized cubes remaining the same like in the experiment and in the other simulations. The data of Fig. S7 shows that for SPB's with a diameter of 2.8 μm the highest transport velocities are expected for the square's edges of some 4–5 μm (compare Ref. [3]). Note that because of the high estimated distance between the PMMA spacer layer and the SPBs's surface (about 1 μm - see the main text) relative to the 10 nm thickness of the (Tb-1.05 nm/Co-0.66 nm)$_6$ magnetic layer, the increase in thickness can be approximated simply by increasing M_{S}. Thus, we expect that by using either a thicker magnetic layer or by having a higher Ms would have a result similar to that of increasing the SPBs's magnetic susceptibility by increasing the forces acting on the beads (see Fig. 5 in the main text), i.e., increasing the critical f_{rot} above which the beads no longer move in a "phase-locked" regime [2].

[1] Ł. Frackowiak, P. Kuświk, G. D. Chaves-O'Flynn, M. Urbaniak, M. Matczak, P. P. Michałowski, A. Maziewski, M. Reginka, A. Ehresmann, and F. Stobiecki, Magnetic domains without domain walls: A unique effect of He^{+} ion bombardment in ferrimagnetic Tb/Co films, Phys. Rev. Lett. **124**, 047203 (2020).

[2] B. B. Yellen, R. M. Erb, H. S. Son, R. Hewlin, Jr., H. Shang, and G. U. Lee, Traveling wave magnetophoresis for high resolution chip based separations, Lab Chip **7**, 1681 (2007).

[3] A. D. Henriksen, N. Rozlosnik, and M. F. Hansen, Geometrical optimization of microstripe arrays for microbead magnetophoresis, Biomicrofluidics **9**, 054123 (2015).

[4] A. Jarosz, D. Holzinger, M. Urbaniak, A. Ehresmann, and F. Stobiecki, Manipulation of superparamagnetic beads on patterned Au/Co/Au multilayers with perpendicular magnetic anisotropy, Journal of Applied Physics **120**, 084506 (2016), https://pubs.aip.org/aip/jap/article-pdf/doi/10.1063/1.4961496/13987077/084506_1_online.pdf.

[5] Q. A. Pankhurst, J. Connolly, S. K. Jones, and J. Dobson, Applications of magnetic nanoparticles in biomedicine, Journal of Physics D: Applied Physics **36**, R167 (2003).

[6] N. Kuleznev, P. A. Polyakov, and V. S. Shevtsov, Theoretical and experimental investigation of the magnetic field of a strongly magnetized permanent magnet, Bulletin of the Russian Academy of Sciences: Physics **82**, 974 (2018).

[7] M. Urbaniak, D. Holzinger, A. Ehresmann, and F. Stobiecki, Magnetophoretic lensing by concentric topographic cylinders of perpendicular magnetic anisotropy multilayers, Biomicrofluidics **12**, 044117 (2018), https://pubs.aip.org/aip/bmf/article-pdf/doi/10.1063/1.5034516/14597607/044117_1_online.pdf.

[8] M. Urbaniak, M. Matczak, G. Chaves-O'Flynn, M. Reginka, A. Ehresmann, and P. Kuświk, Domain wall motion induced magnetophoresis in unpatterned perpendicular magnetic anisotropy Co layers with Dzyaloshinskii-Moriya interactions, Journal of Magnetism and Magnetic Materials **519**, 167454 (2021).

[9] J. Israelachvili, *Intermolecular & Surface Forces* (Academic Press Limited, 1992).

[10] V. A. Hernández, An overview of surface forces and the DLVO theory, ChemTexts **9**, 10 (2023).

[11] A. Ehresmann, I. Koch, and D. Holzinger, Manipulation of superparamagnetic beads on patterned exchange-bias layer systems for biosensing applications, Sensors **15**, 28854 (2015).

[12] N. C. X. Stuhlmüller, F. Farrokhzad, P. Kuświk, F. Stobiecki, M. Urbaniak, S. Akhundzada, A. Ehresmann, T. M. Fischer, and D. de las Heras, Simultaneous and independent topological control of identical microparticles in non-periodic energy landscapes, Nature Communications **14**, 7517 (2023).

[13] FFmpeg Developers, ffmpeg tool, version 4.4.2-0ubuntu0.22.04.1, ffmpeg.org.

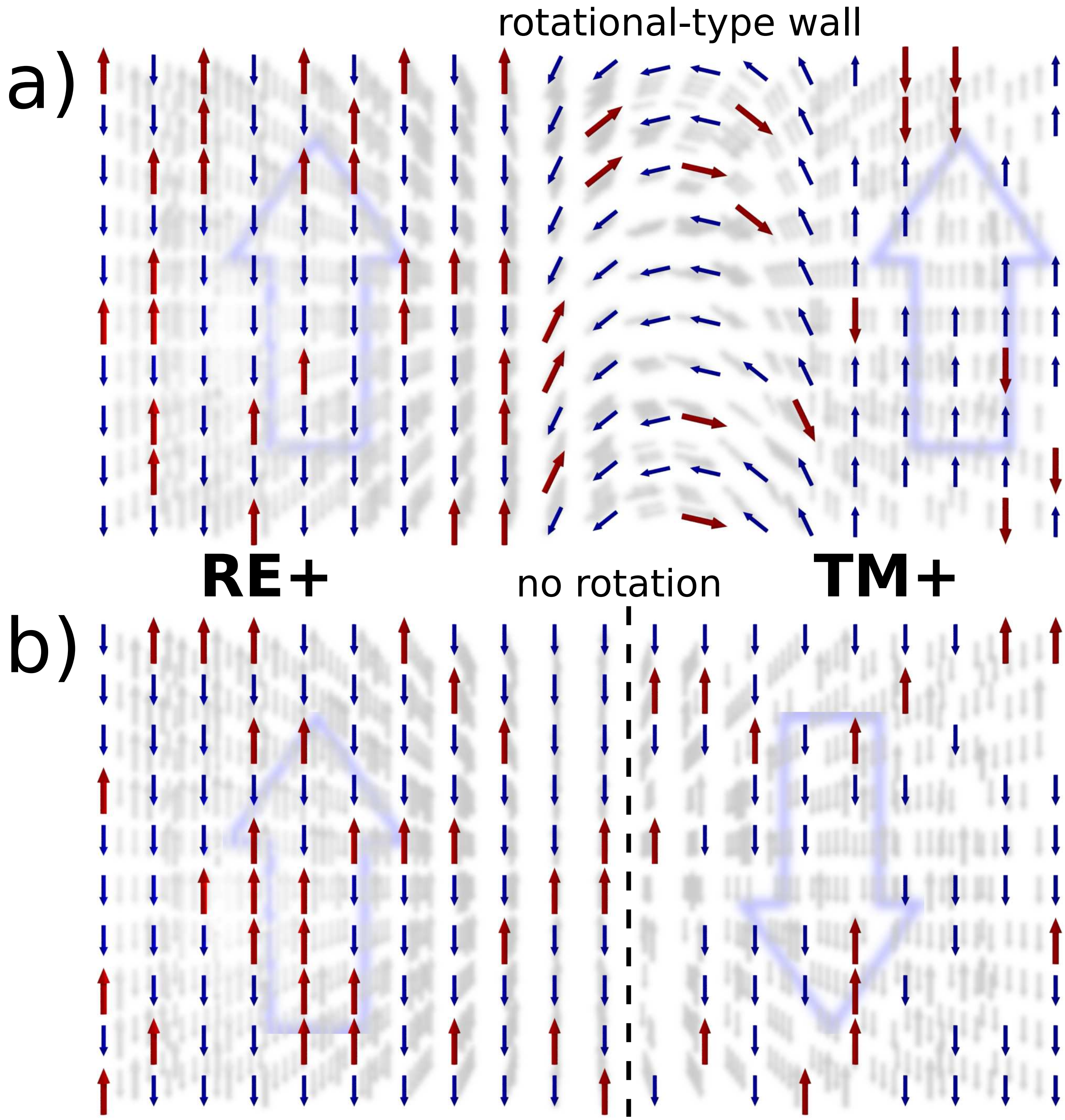

FIG. 1. Schematic of magnetic moments configuration in the Tb/Co multilayer in the vicinity of boundaries between the magnetic domains in the case of parallel (a) and antiparallel (b) orientation of their effective magnetic moments (for the details see Ref. [1]). Note that in the antiparallel case there is no rotational-type domain wall while in (a) the spins within Tb and Co sublattices rotate along the normal to the wall although the magnetization points in the same direction on both its sides.

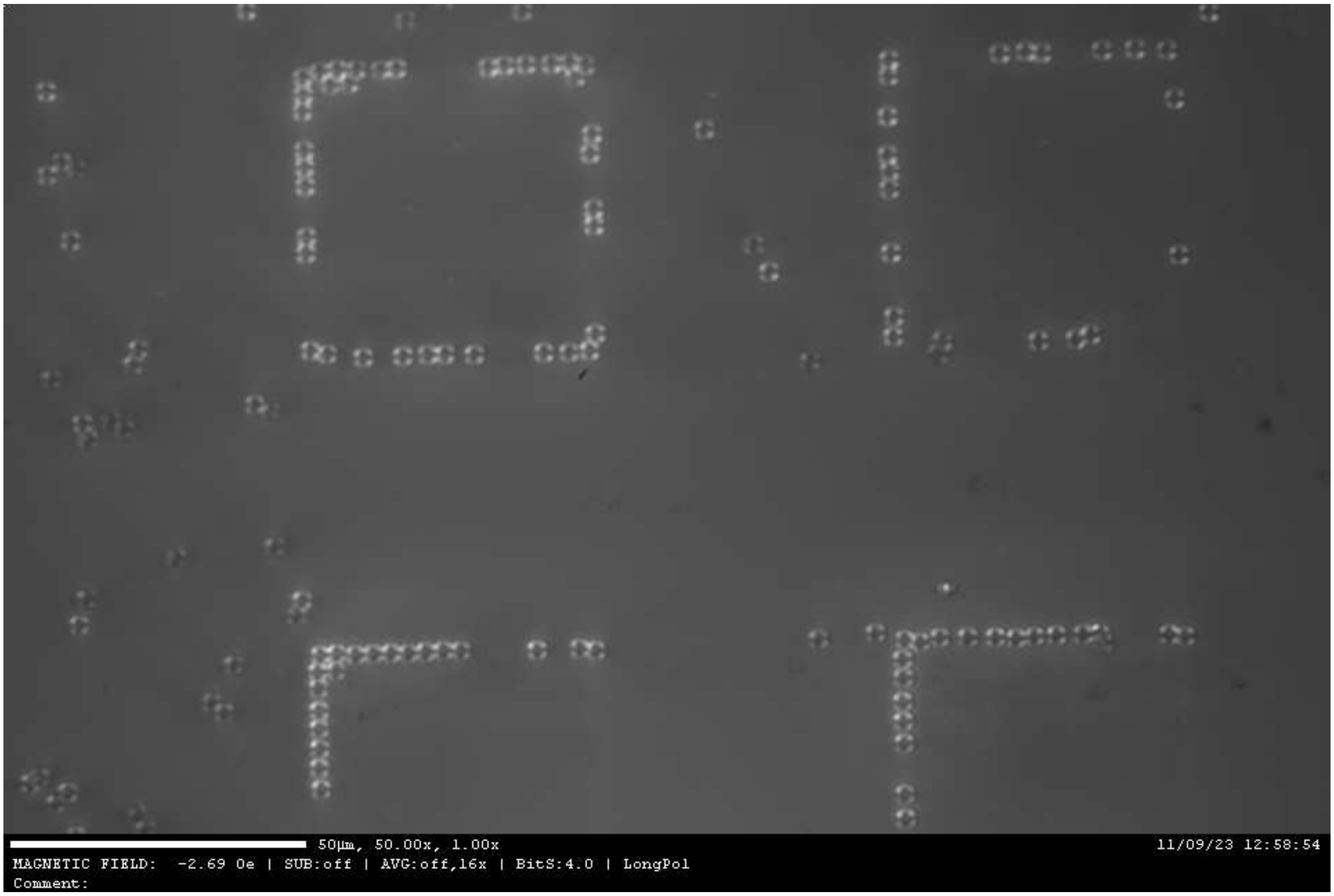

FIG. 2. Image showing the SPBs decorating the domain walls, i.e., regions of high magnetic field strength, of 50×50 μm^2 squares bombarded with 8×10^{13} Ga^{+}/cm^2 ion dose. The $M_{\rm eff}$ of the squares is antiparallel to that of the pristine matrix.

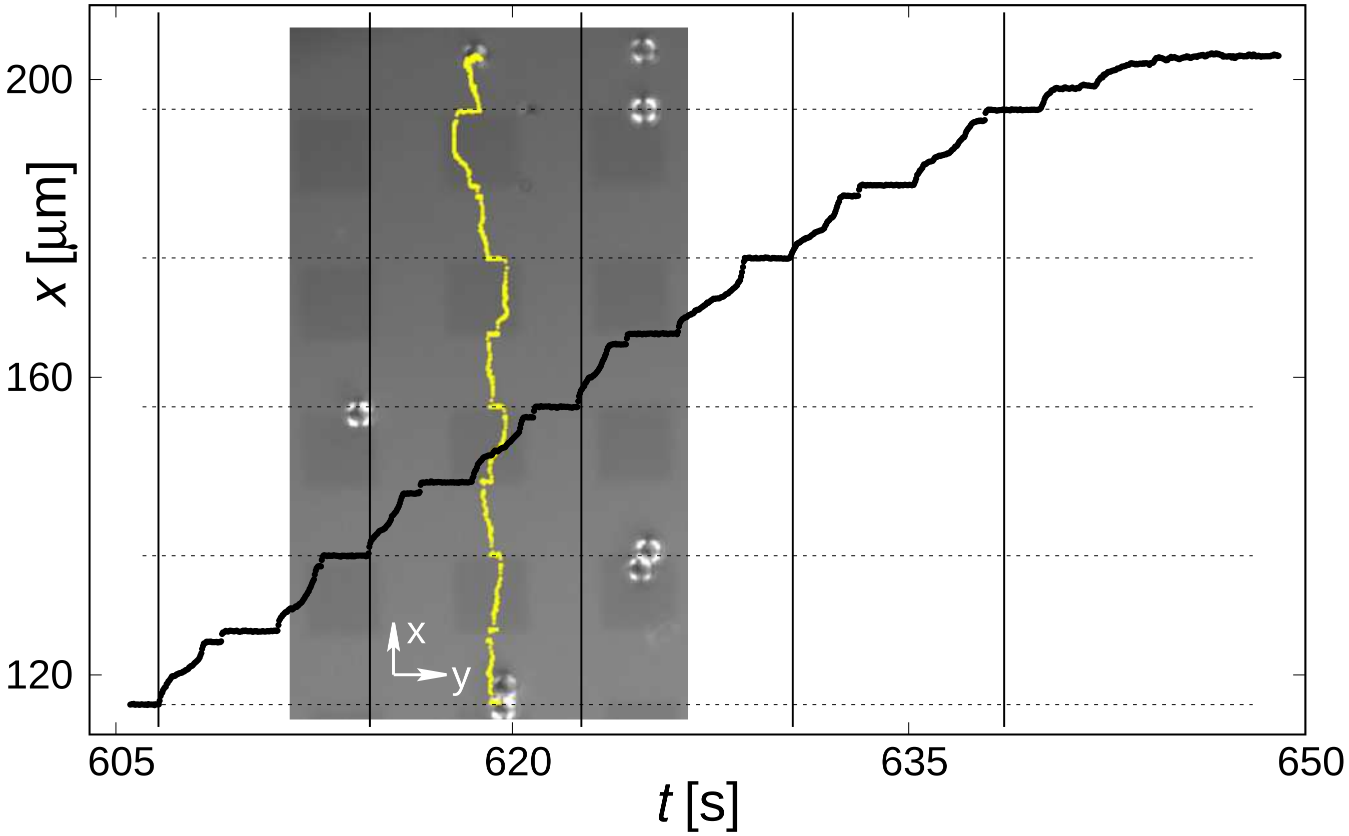

FIG. 3. Exemplary magnetophoretic trajectory of one SPB over the array of 10×10 μm^2 squares bombarded with 15×10^{13} Ga$^+$/cm^2 ion dose. The trajectory is shown as an x-coordinate versus time (black curve) or as a line in the xy-plane (yellow curve). The $M_{\rm eff}$ of the squares is parallel to that of the pristine matrix. The trajectory was recorded with the external field of $\sqrt{2}\times1.59$ kA/m repeatedly switching direction with $f_{\rm rot} = 0.125$ Hz. The field switched by 90° starting with an angle of ±45° to the normal. The vertical lines, spaced by 8 s, correspond to one period of the field cycle ($T_{\rm rot}$). The horizontal dashed line spacing is equal to the the array's lattice spacing. The trajectory was traversed from the bottom to the top.

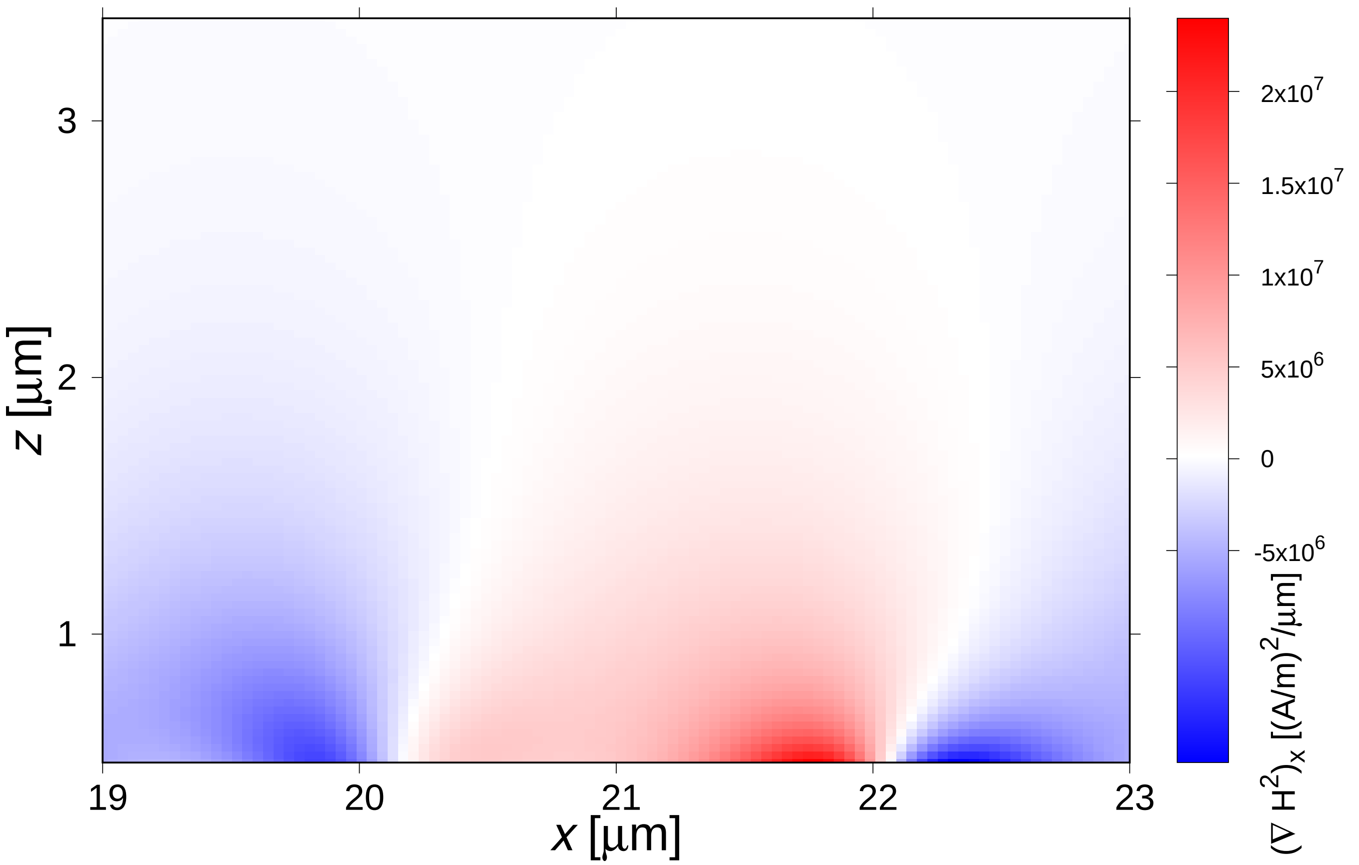

FIG. 4. x-component of the gradient of square the magnetic field strength produced by the array of 11×11, 10 nm thick, square cuboids with $a = 2$ μm, over a central square, in its xz symmetry plane (square edges are at x = 20 μm and x = 22 μm). The squares, embedded in a non-magnetic matrix, are magnetized perpendicularly to the plane of the sample with a constant magnetization of $M_{\mathrm{eff}} = 1$ MA/m. The external field is: $H_x = 1.59$ kA/m, $H_z = $ -1.59 kA/m. The height h relates to the distance from a bottom face of the cuboid. A numerical derivative is calculated using symmetric difference quotient with coordinate changes of 0.0001 μm.

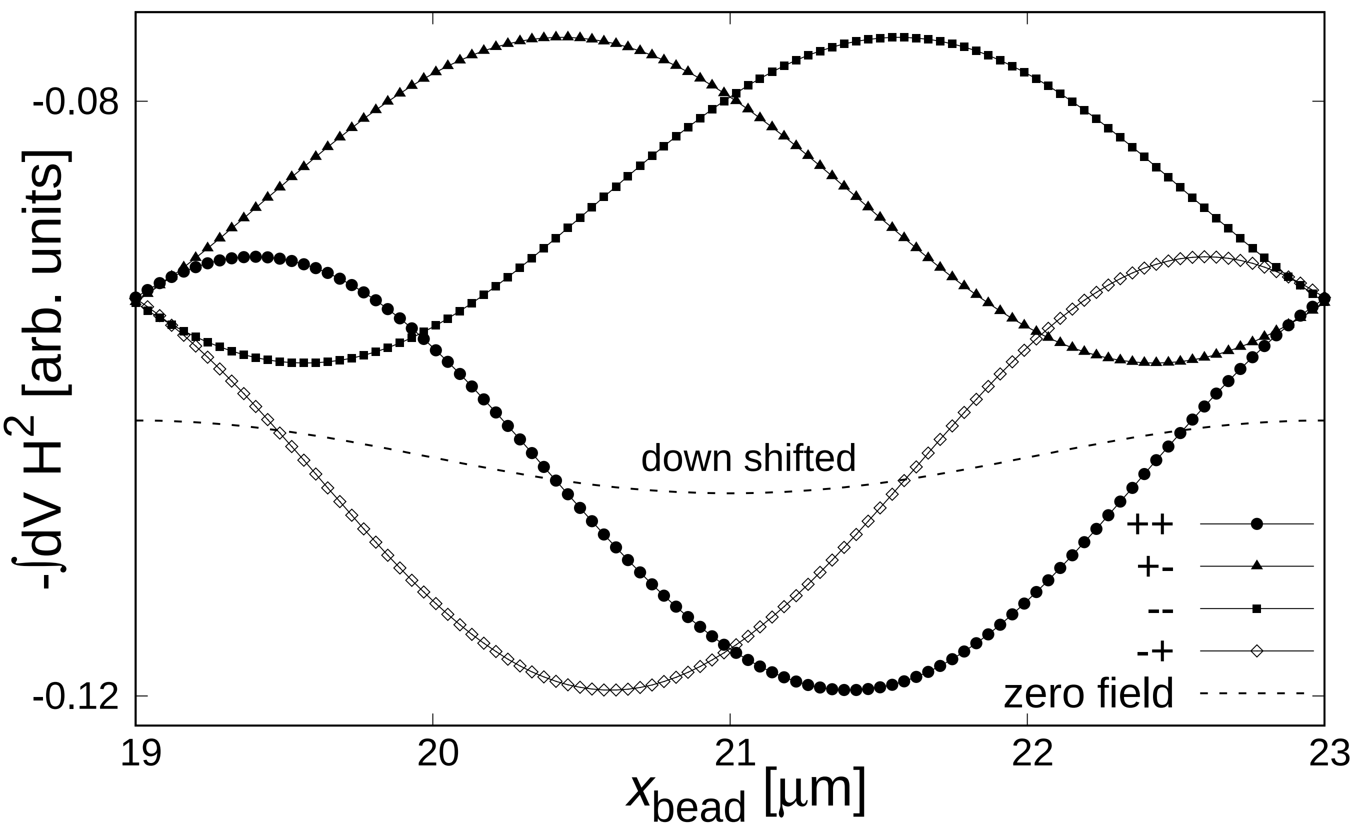

FIG. 5. Exemplary dependencies of the integral of H^2 over the volume of the 2.8 μm diameter bead, with its center in a non-diagonal symmetry plane of the central cuboid and at $z = 2$ μm over the bottom of the magnetic cuboids, as function of the x-position of SPB center; the magnetic structure is the same as described in the caption of Fig. S4. Each curve corresponds to one of the four orientations of $H_{\rm ext}$; the "-+" notation is explained in text.

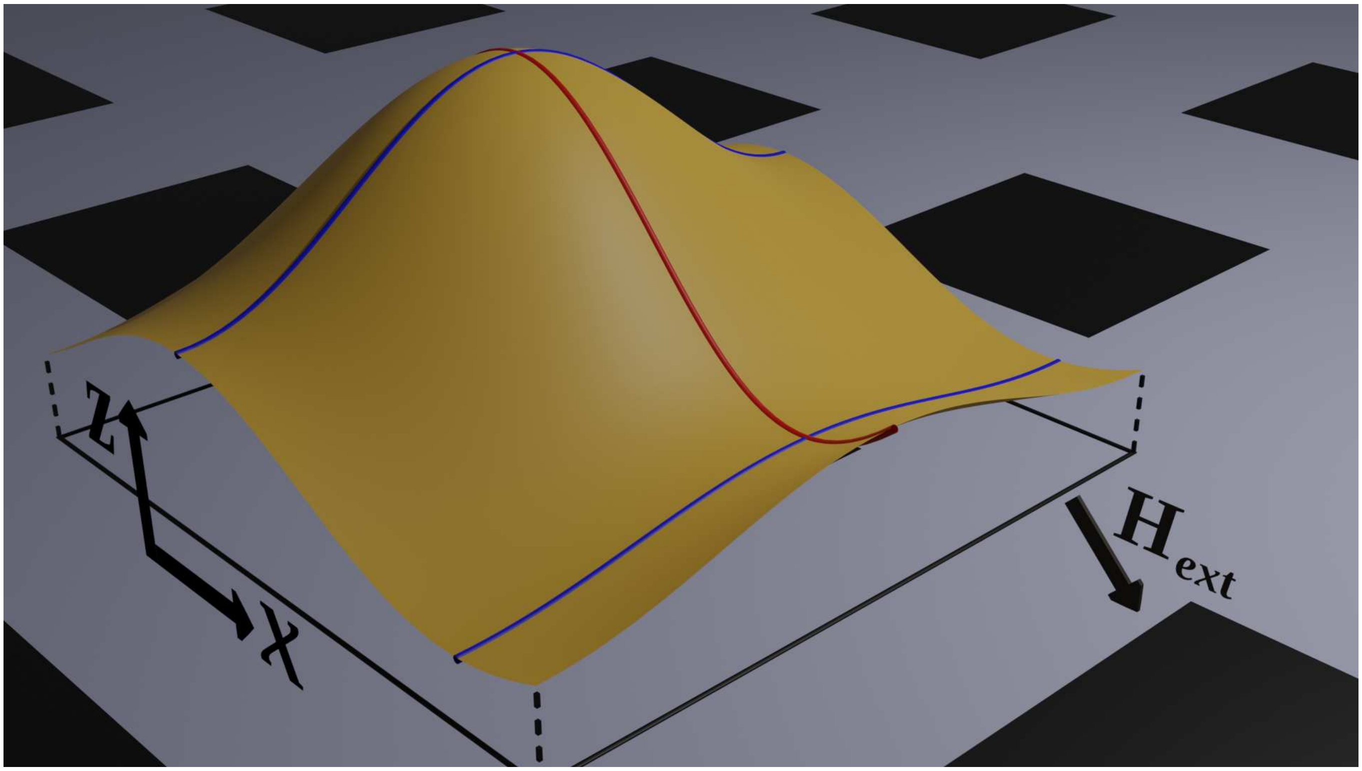

FIG. 6. Exemplary dependence of the integral of $-H^2$ (arb. units) over the volume of the 2.8 μm diameter bead as a function of xy-position of the SPB center. The magnetic structure is the same as described in the caption of Fig. S4, and the yellow surface projection on xy-plane covers one tile of the structure centered on the central cuboid. The center of the bead is fixed at $z = 2$ μm, with the external field of H_x= 1.59 kA/m, and H_z= 1.59 kA/m. The red curve corresponds to the "+-" dependence of Fig. S5.

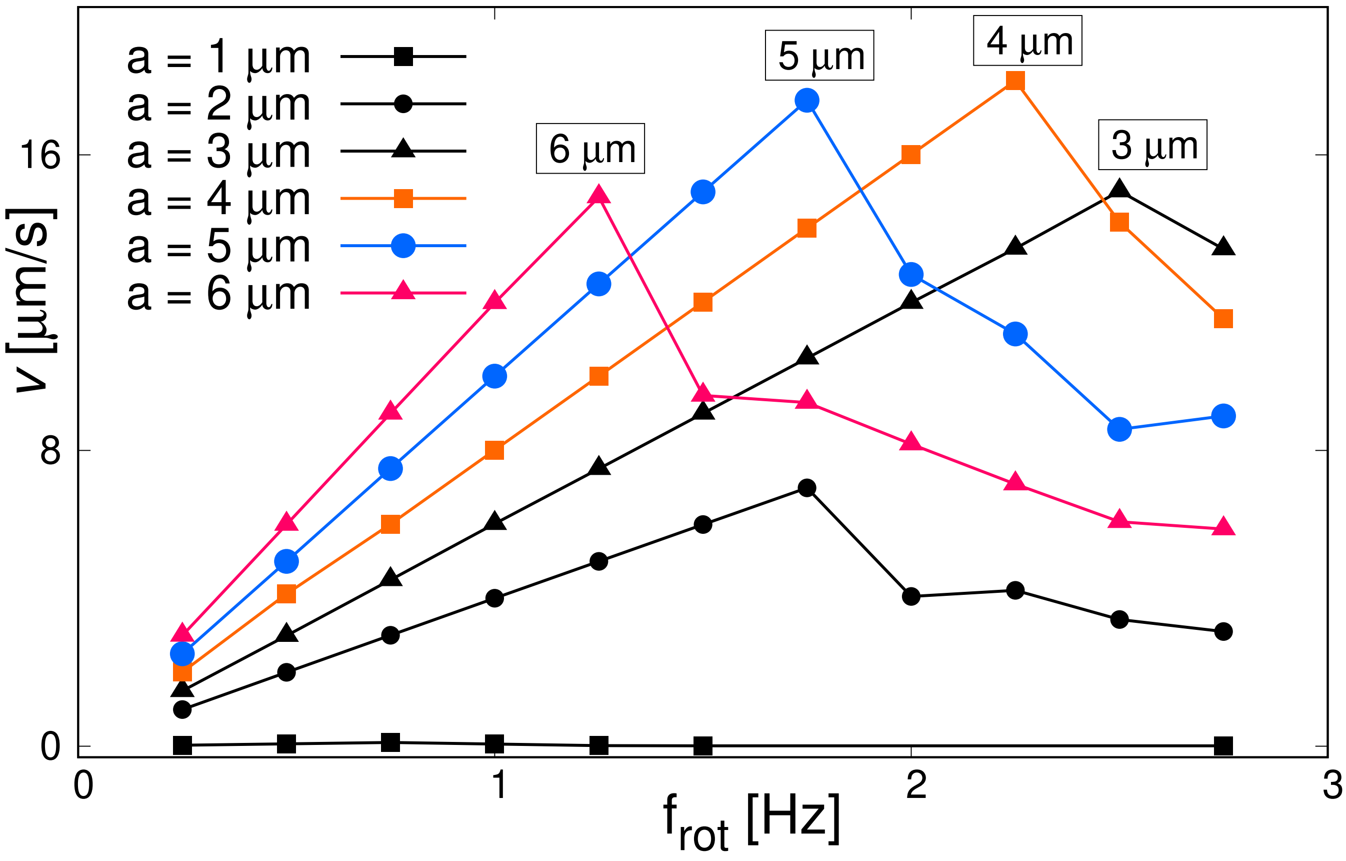

FIG. 7. Simulated dependencies of the 2.8 μm diameter SPBs' velocities on $f_{\rm rot}$ for several in-plane spatial periods of square array, 2·a. The dependence for $a = 2$ μm corresponds to Figs. 5 and 6 of the main text.
